# Structure‐Guided Design of an Interface‐Derived Inhibitor Peptide Against *Spodoptera frugiperda* Digestive Trypsins

**DOI:** 10.1002/arch.70164

**Published:** 2026-05-15

**Authors:** José Severiche‐Castro, Maria Fernanda Valerio, Maria Goreti de Almeida Oliveira

**Affiliations:** ^1^ Facultad de Ciencias Naturales y Exactas Programa de Fisica, Universidad del Valle Cali Colombia; ^2^ Facultad de Educación y Ciencias Programa de Fisica, Universidad de Sucre Sincelejo Colombia; ^3^ Programa de Pós‐graduação em Bioquímica e Biotecnologia, Universidade Federal de Viçosa Viçosa Brazil

**Keywords:** armyworm, binding free energy, interface, MM/GBSA, molecular dynamics

## Abstract

*Spodoptera frugiperda* is a major agricultural pest whose control requires safer and more sustainable alternatives. In this study, a rational in silico strategy was applied to design a bioinsecticidal peptide targeting digestive trypsin from *S. frugiperda*, using a functionally relevant interfacial region of the Dioscorin–trypsin complex as a structural template. Interface analysis allowed the identification of an 11‐residue oligopeptide designated as PEP‐11. Trypsin–peptide docking, pharmacophore profiling, and triplicate molecular dynamics simulations were performed to characterize its interaction with the trypsin model. Docking analysis revealed that PEP‐11 binds through a cooperative network of polar and hydrophobic interactions, supporting favorable molecular recognition. Molecular dynamics analyses showed that the trypsin–PEP‐11 complex remained stable throughout the 100 ns simulations, with limited structural deviations and flexibility mainly restricted to terminal and loop‐exposed regions. In addition, the radius of gyration and solvent‐accessible surface area remained stable, while intermolecular hydrogen bonds increased over time. MM/GBSA calculations yielded negative binding free energy values in all replicas, indicating energetically favorable binding. Overall, these results support the computational prioritization of PEP‐11 as an interface‐derived peptide candidate for future experimental evaluation, including enzymatic inhibition, selectivity, proteolytic stability, and bioactivity assays.

## Summary

- PEP‐11 was designed from hot‐spot residues at the Dioscorin–trypsin interface.
- Docking supported favorable recognition of PEP‐11 by S. frugiperda trypsin.
- Triplicate molecular dynamics simulations showed stable peptide binding.

## Graphical abstract

In this study, a rational *in silico* strategy was employed to design a bioinsecticidal peptide targeting the digestive trypsins of *Spodoptera frugiperda*, utilizing the Dioscorin–trypsin interface as a structural template. By identifying critical interface hot‐spot residues, an 11‐residue oligopeptide designated as PEP‐11 was engineered, demonstrating favorable molecular recognition through a cooperative network of polar and hydrophobic interactions in docking analyses. Triple 100 ns molecular dynamics simulations confirmed the stability of the complex, evidenced by minimal structural deviations (RMSD), consistent compactness (Radius of Gyration), and a progressive increase in intermolecular hydrogen bonds. Furthermore, MM/GBSA calculations yielded consistently negative binding free energy values, computationally validating PEP‐11 as a promising scaffold for the development of sustainable, peptide‐based biotechnological alternatives for agricultural pest control.

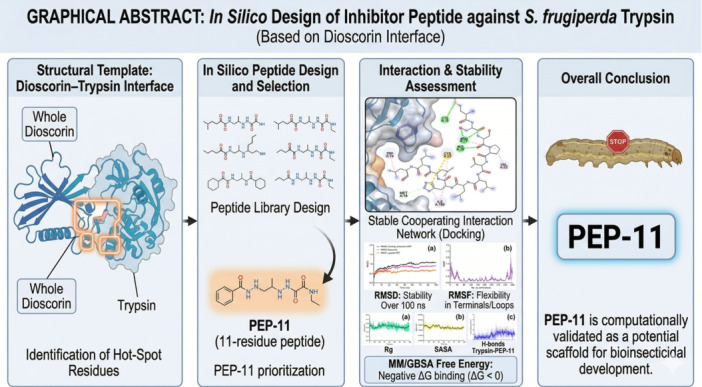

## 1 Introduction

In the field of pest management research, the development of novel insecticidal agents that are both safe and effective is of critical importance for addressing challenges associated with crop protection and public health (Al Naggar et al. 2025). Insect pests such as *Spodoptera frugiperda* pose a major threat to economically important crops, including maize, rice, sorghum, sugarcane, cotton, and several horticultural species, thereby driving the search for innovative and sustainable control strategies. The damage caused by *S. frugiperda* is primarily associated with larval feeding on leaves, stems, and plant whorls, which can result in substantial reductions in both crop yield and quality.

Molecular modeling has emerged as a valuable strategy in peptide‐based drug discovery. This computational approach enables the prediction and characterization of the interactions of peptides or proteins with their molecular targets, including enzymes, receptors, ion channels, and other biologically relevant macromolecules (Le et al. 2024). Such peptides may be either naturally occurring or rationally designed, and the main goal is to identify or engineer protein‐derived molecules with specific biological activity and high affinity for their targets, thereby making them promising candidates for therapeutic or biotechnological applications.

In this context, the rational design of bioinsecticidal peptides through molecular dynamics simulations represents a promising approach for developing molecules capable of inhibiting specific proteases in insect pests. This strategy involves the use of computational techniques to simulate the interaction between a peptide inhibitor and a target protein in order to understand both the molecular basis of binding and the dynamic behavior of the complex over time. Rational peptide design based on the trypsin–inhibitor interaction has attracted particular interest because of the crucial functional role of trypsin‐like proteases in the digestive system of herbivorous insects such as *S. frugiperda*. These enzymes are responsible for the hydrolysis of dietary proteins into amino acids essential for growth and development. Conversely, peptide inhibitors are capable of binding to and blocking trypsin activity, thereby potentially disrupting digestive processes and ultimately impairing insect development and survival.

Protein–protein interactions (PPIs) are widely recognized as a promising class of molecular targets in drug discovery and, in the present case, in bioinsecticide development, due to their central involvement in a broad range of biological processes. The present study focuses on the rational design of peptides derived from the trypsin–Dioscorin inhibitor interaction, a protein–protein complex previously adopted as a model system in our research line (Severiche‐Castro et al. 2023). The interaction between Dioscorin, an inhibitor protein from Dioscorea alata, and insect trypsins provides an example of a biologically relevant PPI with potential application in pest control. However, the direct use of full‐length proteins as inhibitors in agricultural settings is constrained by high production costs, large molecular size, and limited environmental stability. Therefore, the aim of this study was to explore the feasibility of developing an interface‐derived peptide with favorable predicted binding toward digestive trypsins of *S. frugiperda*, while providing structural information that may guide future selectivity assessment against non‐target proteases.

These peptides have the potential to become promising candidates for the development of biological insecticides, as they are expected to be less harmful to non‐target organisms and the environment. In addition, due to their small size, these peptides may be easier to produce, handle, and scale up than full‐length proteins, which could facilitate their future implementation in agricultural pest control strategies. In the present study, computational tools such as molecular modeling, protein–peptide docking, and molecular dynamics simulations were employed to design and evaluate the interaction between the peptide and trypsin. Furthermore, key binding regions were structurally analyzed, and the amino acid residues responsible for specific interactions were identified.

Rather than being viewed merely as a preliminary computational exercise, this study should be understood as a rational screening and design platform for the identification of bioinsecticidal peptides with potential experimental value. By integrating structural analysis, docking, and molecular dynamics simulations, this approach enables the recognition of key binding determinants and the prioritization of candidate sequences before synthesis and biological evaluation. Therefore, this work provides a mechanistic and structural framework that may guide subsequent in vitro, in vivo, and formulation studies aimed at developing peptide‐based bioinsecticides against *S. frugiperda*.

## 2 Materials and Methods

### 2.1 In Silico Strategy for the Peptide Design

Based on our previous structural study of the interaction between digestive trypsins from *S. frugiperda* and the yam protein Dioscorin (Severiche‐Castro et al. 2023), Dioscorin was identified as a promising inhibitor due to its high binding potential toward insect trypsins and its predicted ability to reduce their proteolytic activity. The validated three‐dimensional model of *S. frugiperda* digestive trypsin used in the present study was also derived from that previous work. Briefly, trypsin sequences were obtained from translated genes previously identified by zymogram analysis and mass spectrometry, and their domain architecture was verified using the Pfam database. Structural prediction was performed with the Robetta server, and model quality was assessed through PRoSA, ERRAT, and Ramachandran plot analysis. The validated trypsin model selected from that study was used here as the receptor for peptide docking and molecular dynamics simulations.

The docked complex between *S. frugiperda* trypsin and Dioscorin was obtained from previous protein–protein docking analyses performed using the ClusPro server (Kozakov et al. 2017). ClusPro performs rigid‐body docking, followed by clustering of the lowest‐energy conformations and refinement through CHARMM‐based energy minimization, generating ten representative models as output. The best‐ranked complex was selected for interfacial analysis based on its clustering and energy criteria.

The protein–protein interface was analyzed to identify Dioscorin residues involved in complex formation. Interacting residues were defined using a distance cutoff of less than 4 Å between the two protein chains, followed by structural inspection in PyMOL (The PyMOL Molecular Graphics System, Version 2.0, Schrödinger LLC). The selected residues were therefore defined as functionally relevant interfacial residues, considering their close contact with trypsin, spatial continuity within the Dioscorin interface, and contribution to the interaction pattern of the parent complex. Particular emphasis was placed on the Dioscorin interfacial loop previously identified by Severiche‐Castro et al. (2023), which is positioned near the trypsin binding surface and contributes substantially to active‐site blockade.

The peptide design strategy was therefore focused on extracting a short contiguous sequence from this interface‐derived region while preserving the residues involved in direct contacts with trypsin and maintaining the local interaction pattern of the parent complex. Candidate residues were prioritized according to their participation in intermolecular contacts, their energetic contribution within the interface, and their spatial continuity along the Dioscorin sequence. Based on these criteria, the 11‐residue peptide MSSPTLLHLLL was selected as the final candidate, hereafter designated as PEP‐11, for subsequent protein–peptide docking and molecular dynamics analyses.

### 2.2 Protein–Peptide Docking and Molecular Dynamics Simulations

Protein–peptide docking was performed using the ClusPro PeptiDock server (Porter et al. 2017) to predict the binding mode of PEP‐11 within the S. frugiperda trypsin model. Prior to docking, the receptor structure was prepared by removing non‐essential molecules, adding hydrogen atoms, and verifying the structural integrity of the binding region. The peptide was submitted as an independent ligand structure and docked against the trypsin model.

The docking search was focused on the trypsin surface corresponding to the Dioscorin‐interacting region identified in the parent Dioscorin–trypsin complex, in order to preserve the structural rationale of the interface‐derived design. In this protocol, the receptor was treated as rigid, whereas the peptide was allowed to explore alternative orientations within the selected interfacial region. A total of ten docking poses were generated and ranked according to the clustering and scoring criteria implemented in ClusPro PeptiDock.

The final pose selected for subsequent pharmacophore profiling and molecular dynamics simulations corresponded to one of the top‐ranked solutions located within the predefined interfacial region. This pose was selected because it preserved the expected interface‐guided binding orientation and displayed a chemically coherent interaction network, including hydrogen bonds and hydrophobic contacts with residues positioned near the trypsin binding surface. The selected trypsin–PEP‐11 complex was then analyzed in Discovery Studio Visualizer (BIOVIA 2019) to identify the main non‐covalent interactions contributing to complex stabilization.

After solvation and ion addition, each system was subjected to energy minimization for 1000 steps to remove unfavorable contacts. The minimized systems were then gradually equilibrated before production dynamics. First, short restrained equilibration was performed under NVT conditions to stabilize temperature, followed by NPT equilibration at 310 K and 1 atm to allow solvent density and box dimensions to adjust. The production simulations were then carried out for 100 ns under NPT conditions.

All simulations were performed using a 2 fs integration time step. Covalent bonds involving hydrogen atoms were constrained, and trajectory coordinates were saved every 10 ps for subsequent analysis. Short‐range non‐bonded interactions were calculated using a 12 Å cutoff, whereas long‐range electrostatic interactions were treated using the particle mesh Ewald method. Temperature was controlled using Langevin dynamics, and pressure was maintained using the Langevin piston method.

To evaluate reproducibility, three independent molecular dynamics replicas were performed under identical physical conditions but initiated with different initial velocity distributions. These independent starting velocities allowed each replica to explore the conformational space from the same docked complex through distinct dynamical trajectories. The final ionic composition of the simulated system included sodium, chloride, and calcium ions added using the Autoionize plugin implemented in VMD to neutralize the system and ensure electrostatic stability of the simulation system.

### 2.3 Trajectory Analysis and Binding Free Energy Estimation

All molecular dynamics simulations of the trypsin–PEP‐11 complex were carried out in triplicate as three independent replicas under identical conditions. Post‐simulation analyses were performed for each trajectory, and the reported profiles correspond to the average behavior of the three replicas. When indicated, the solid line represents the mean value and the surrounding shaded area corresponds to the standard deviation.

Trajectory analyses were performed to evaluate the structural stability, compactness, solvent exposure, flexibility, and intermolecular interactions of the complex throughout the simulation. Prior to analysis, all frames were aligned to the initial structure using the backbone atoms of the receptor in order to remove global rotational and translational motions.

The root mean square deviation (RMSD) was calculated as a function of simulation time for the whole complex, the receptor, and the peptide ligand, in order to monitor their conformational stability during the trajectory. The root mean square fluctuation (RMSF) was calculated on a per‐residue basis using the Cα atoms of the receptor to identify the most flexible regions of the protein.

The radius of gyration (Rg) was calculated to assess the global compactness of the complex during the simulation, whereas the solvent‐accessible surface area (SASA) was determined to evaluate changes in solvent exposure over time. In addition, the number of intermolecular hydrogen bonds between the receptor and the peptide was monitored throughout the trajectory using geometric criteria based on donor–acceptor distance and bond angle. These analyses allowed the identification of the main structural features associated with complex stability and peptide binding persistence.

### 2.4 Binding Free Energy Calculation

The binding free energy of the trypsin–PEP‐11 complex was estimated using the molecular mechanics generalized Born surface area (MM/GBSA) approach, following the strategy previously applied in our structural study of the Dioscorin–trypsin system. MM/GBSA calculations were performed with the MOLAICAL software, which enables binding free energy estimation from molecular dynamics trajectories generated with NAMD. For MM/GBSA calculations, snapshots were extracted from the equilibrated production phase of each 100 ns trajectory. To minimize the influence of the initial structural relaxation stage, only frames from the final 50 ns of each simulation, corresponding to the 50–100 ns interval, were considered. A total of 100 snapshots per replica were selected at regular 0.5 ns intervals and used for binding free energy estimation.

The binding free energy values reported for each replica correspond to the mean ± standard deviation obtained from the analyzed snapshots. Electrostatic and van der Waals energy components were also extracted and reported as average values. The reported values were interpreted as relative energetic estimates useful for comparing the consistency and favorability of peptide binding across independent replicas, rather than as absolute predictors of experimental affinity. The binding free energy was calculated according to:

$$\Delta G_{\text{bind}}=G_{\mathrm{RL}}-G_{R}-G_{L}$$

where $G_{\mathrm{RL}}$, $G_{R}$, and $G_{L}\;$correspond to the free energies of the complex, the isolated receptor, and the isolated ligand, respectively.

In MM/GBSA formalism, the binding free energy can also be expressed as:

$$\Delta G_{\text{bind}}=\Delta H-T\Delta S=\Delta E_{\text{MM}}+\Delta G_{\text{sol}}-T\Delta S$$

where $\Delta E_{\text{MM}}$represents the gas‐phase molecular mechanics energy, $\Delta G_{\text{sol}}$is the solvation free energy, and TΔSis the entropic contribution. The molecular mechanics term includes electrostatic $\left(\Delta E_{\text{ele}}\right)$, van der Waals $\left(\Delta E_{\text{vdW}}\right)$, and internal $\left(\Delta E_{\mathrm{int}}\right)$ energy contributions, whereas the solvation term includes polar solvation, calculated using the generalized Born model, and nonpolar solvation, estimated from the solvent‐accessible surface area. In the present study, the entropic term was neglected in order to reduce computational cost, as only relative binding energies were considered.

For the MM/GBSA calculations, a solvent dielectric constant of 78.5 and a surface tension constant of 0.03012 kJ mol⁻¹ Å⁻² were used. In addition to the total binding free energy, electrostatic and van der Waals energy components were extracted from the analyzed snapshots and reported as average values for each independent simulation. Binding free energy values are presented as mean ± standard deviation.

## 3 Results

### 3.1 In Silico Strategy for the Peptide Design

Structural analysis of the Dioscorin–trypsin interface led to the identification of an 11‐residue oligopeptide, MSSPTLLHLLL (Met–Ser–Ser–Pro–Thr–Leu–Leu–His–Leu–Leu–Leu), which was selected as the peptide candidate and is hereafter referred to as PEP‐11. From a compositional standpoint, the peptide contains an N‐terminal region enriched in polar uncharged residues, including serine and threonine, as well as a proline residue that may influence local backbone conformation.

In contrast, the central and C‐terminal regions are predominantly hydrophobic due to the high leucine content, with a histidine residue that may contribute to specific interactions depending on the binding microenvironment. Overall, this amino acid composition suggests that the peptide exhibits a moderately amphipathic character, which may favor both molecular recognition and stabilization within the trypsin binding region.

**Figure 1:**
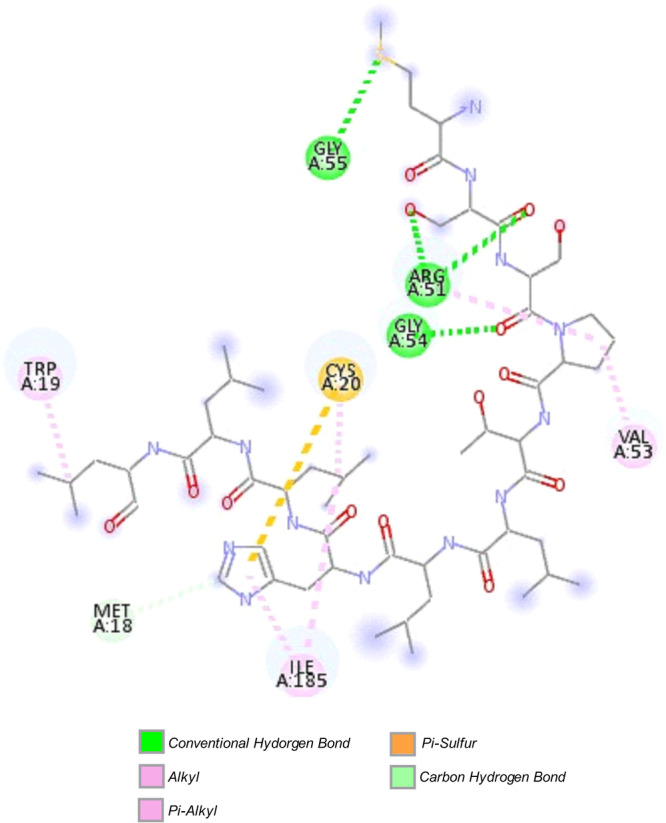
Two‐dimensional representation of the molecular interactions between the designed peptide PEP‐11 and the trypsin model of *Spodoptera frugiperda*. The complex is stabilized by conventional hydrogen bonds, alkyl and π‐alkyl hydrophobic contacts, as well as π‐sulfur and carbon–hydrogen interactions involving the residues Met18, Trp19, Cys20, Arg51, Val53, Gly54, Gly55, and Ile185.

Docking analysis showed that PEP‐11 interacts with the *S. frugiperda* trypsin model through a structurally coherent and chemically diverse interaction network. As illustrated in Figure 1, the complex is primarily stabilized by conventional hydrogen bonds involving Arg51, Gly54, and Gly55, which likely act as key anchoring residues by interacting with oxygen‐containing groups of PEP‐11. These polar contacts are complemented by hydrophobic alkyl and π‐alkyl interactions involving Trp19, Val53, and Ile185, which may enhance peptide accommodation and binding stability within the receptor environment.

Additional contributions from a π‐sulfur interaction with Cys20 and a carbon–hydrogen bond with Met18 further support the formation of a stable trypsin–PEP‐11 complex. Taken together, these results indicate that peptide binding is not governed by a single interaction type, but rather by a cooperative balance of hydrogen‐bonding and hydrophobic contacts, highlighting Met18, Trp19, Cys20, Arg51, Val53, Gly54, Gly55, and Ile185 as the main residues involved in molecular recognition.

### 3.2 Trajectory Analysis and Binding Free Energy Estimation

**Figure 2:**
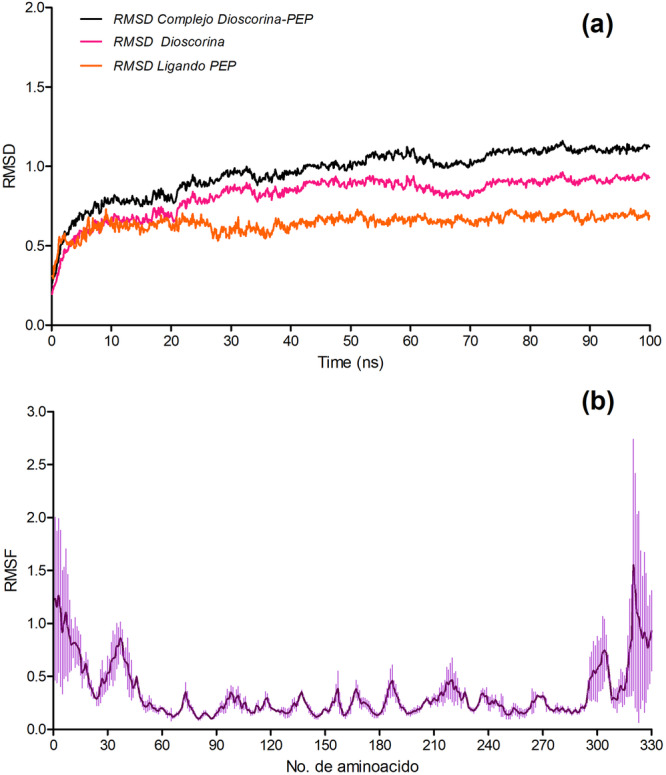
Molecular dynamics stability analysis of the trypsin–PEP‐11 complex based on triplicate simulations. (a) RMSD profiles of the complex, trypsin, and PEP‐11 during the 100 ns simulation. (b) RMSF profile of trypsin residues. Curve represent the mean values obtained from three independent simulations, and shaded regions indicate the corresponding standard deviations.

The dynamic stability of the trypsin–PEP‐11 complex was investigated through three independent molecular dynamics simulations. The reported profiles correspond to the mean behavior of the replicas, with standard deviations shown when applicable. To characterize the conformational behavior of the complex, trajectory analyses including RMSD and RMSF were performed (Figure 2).

As shown in Figure 2a, the RMSD profile of the complex exhibited a rapid increase during the first 10 ns, consistent with the initial relaxation of the system from the starting docked conformation. After this equilibration stage, the complex showed a more gradual increase and then remained relatively stable, fluctuating around 0.9–1.1 nm throughout the remainder of the 100 ns simulation. This behavior indicates that the complex preserved its overall structural integrity without evidence of major conformational disruption.

A similar trend was observed for trypsin alone, which showed an initial increase followed by stabilization around 0.8–0.95 nm. In contrast, PEP‐11 displayed lower RMSD values during the entire trajectory, remaining mostly within the range of 0.55–0.70 nm. The lower fluctuation of the peptide suggests that, once accommodated in the binding region, PEP‐11 maintained a relatively stable conformation throughout the simulation. Altogether, these results support the dynamic stability of the trypsin–PEP‐11 complex under the simulated conditions.

The residue‐level flexibility of trypsin was further assessed by RMSF analysis (Figure 2b). Most amino acid residues showed low fluctuation values, generally within the range of 0.1–0.5 nm, indicating preservation of the overall protein fold during the simulation. The highest fluctuations were detected at the N‐terminal and C‐terminal regions, with additional peaks in a few loop‐exposed segments, particularly around residues 25–45 and 300–325.

These fluctuations are likely associated with terminal and solvent‐exposed flexible regions rather than with destabilization of the protein core. Thus, the RMSF profile indicates that the major conformational mobility was restricted to localized segments, whereas the central structural framework of trypsin remained comparatively rigid.

**Figure 3:**
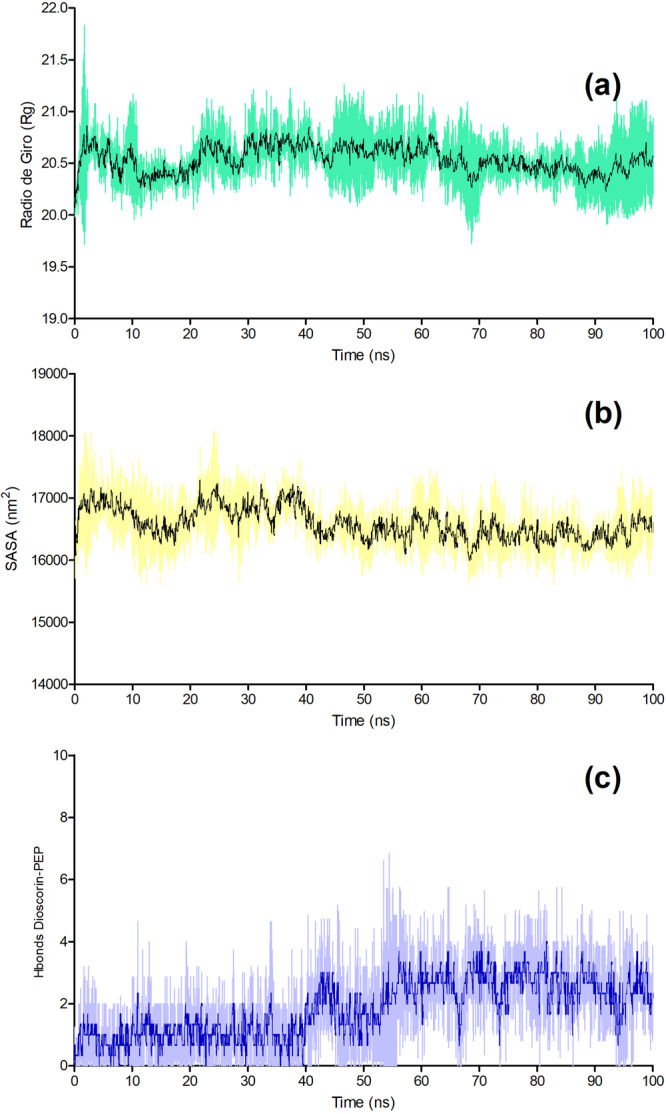
(a) Radius of gyration (Rg) profile. (b) Solvent‐accessible surface area (SASA) profile. (c) Number of intermolecular hydrogen bonds between trypsin and PEP‐11. In all panels, the central line represents the mean value and the shaded region indicates the standard deviation among the three independent simulations.

The structural behavior of the trypsin–PEP‐11 complex was further characterized by Rg, SASA, and the number of intermolecular hydrogen bonds throughout the 100 ns molecular dynamics simulations performed in triplicate (Figure 3). The reported profiles correspond to the mean behavior of the replicas, while the shaded regions indicate the corresponding standard deviations.

As shown in Figure 3a, the Rg profile remained relatively stable during the simulation, fluctuating within a narrow range around 20.3–20.8 Å. Although small oscillations were observed along the trajectory, no pronounced shifts were detected, indicating that the overall compactness of the trypsin–PEP‐11 complex was preserved over time. This behavior suggests the absence of major structural expansion or collapse during the simulation.

A comparable trend was observed for SASA (Figure 3b), which exhibited moderate fluctuations but remained overall stable throughout the trajectory. After the initial stages of the simulation, SASA values showed a slight tendency to decrease and then remained within a relatively consistent range, suggesting minor rearrangements in solvent exposure without evidence of substantial conformational destabilization. Together with the Rg results, this profile supports the maintenance of a structurally coherent complex during the simulation time.

The intermolecular hydrogen bond analysis revealed a progressive increase in the number of hydrogen bonds established between trypsin and PEP‐11 along the trajectory (Figure 3c). During the initial phase of the simulation, the complex exhibited a relatively low number of hydrogen bonds, generally around 0–1. However, after approximately 40–50 ns, the number of intermolecular hydrogen bonds increased and remained mostly within the range of 2–4 until the end of the simulation, with occasional higher peaks. This behavior indicates that peptide binding became more stabilized over time, likely as a result of improved accommodation of PEP‐11 within the trypsin binding region.

The energetic analysis of the trypsin–PEP‐11 complex showed a highly consistent binding pattern across the three independent molecular dynamics simulations (Table 1). The average electrostatic energy remained very similar among replicas, ranging from −7.019 to −7.045 kcal/mol, while the average van der Waals contribution also showed minimal variation, with values between −1.173 and −1.190 kcal/mol. This consistency indicates that the main intermolecular energetic contributions were reproducible throughout the independent simulations.

**Table 1:** Energy components and binding free energy obtained for the trypsin–PEP‐11 complex from the three independent molecular dynamics simulations.

Simulation	Average electrostatic energy (kcal/mol)	Average VdW energy (kcal/mol)	Binding free energy, ΔG (kcal/mol)
1	−7.031	−1.178	−22.9132 ± 0.2609
2	−7.019	−1.173	−23.3232 ± 0.4312
3	−7.045	−1.190	−22.0516 ± 0.1940

*Note:* Binding free energy values are reported as mean ± standard deviation from the analyzed trajectory frames, whereas electrostatic and van der Waals energies are presented as average values.

The estimated binding free energy ΔGwas negative in all three simulations, confirming the energetic favorability of PEP‐11 binding to trypsin. Among the replicas, Simulation 2 exhibited the most favorable binding free energy, with a value of −23.3232±0.4312kcal/mol. Although slight differences were observed among simulations, the relatively narrow range of ΔGvalues suggests a stable and reproducible interaction profile for the trypsin–PEP‐11 complex.

## 4 Discussion

The present study supports the utility of a structure‐guided strategy for deriving short peptide candidates from functionally relevant interfacial regions of protein–protein interaction templates targeting digestive trypsins from *S. frugiperda*. Although protein–protein interfaces are structurally complex, selected interfacial segments may retain key molecular recognition determinants and therefore serve as rational templates for peptide design. In this framework, PEP‐11 is proposed as an interface‐derived peptide candidate that preserves relevant features of the Dioscorin–trypsin binding region and provides a focused scaffold for subsequent experimental validation (Q. Froes and S. Castilho 2024; Meriño‐Cabrera et al. 2020; Ibarra et al. 2019).

In that sense, the relevance of PEP‐11 lies not only in its computational identification, but in its ability to capture a minimal interfacial segment that may preserve the recognition logic of the parent Dioscorin–trypsin complex. This interpretation is consistent with the broader concept of structure‐guided PPI inhibitor design, in which short peptides are used to reproduce interfacial side‐chain organization while avoiding some of the practical limitations associated with full‐length protein inhibitors (Wang et al. 2021; Pelay‐Gimeno et al. 2015). Although these precedents come mainly from biomedical PPI inhibitor design, the same conceptual framework is applicable here: if a restricted set of interfacial residues concentrates the recognition determinants of the Dioscorin–trypsin complex, then a short peptide derived from that region may retain meaningful binding information while offering clear advantages in size, tractability, and future optimization.

The sequence features of PEP‐11 help explain why this fragment emerged as a plausible binder rather than an arbitrary linear segment. Its N‐terminal region contains polar uncharged residues that are well suited to establish directional contacts, whereas the Leu‐rich central/C‐terminal region provides a hydrophobic surface that can favor interfacial packing. This type of compositional asymmetry is often advantageous in interface‐derived peptides because effective recognition commonly depends on combining anchoring polar interactions with a hydrophobic face that contributes to affinity (Fu et al. 2026; Mao et al. 2024). In addition, the presence of Pro may restrict local conformational freedom, which can reduce the entropic penalty of binding, while His may add context‐dependent versatility because its imidazole side chain can switch between more hydrophobic and more polar behavior depending on the local environment (Calinsky and Levy 2024). These features support the idea that PEP‐11 is not an arbitrary linear fragment, but a sequence with physicochemical properties compatible with compatible with stable interfacial binding.

The interaction pattern observed for PEP‐11 is also meaningful from a mechanistic standpoint. Instead of relying on a single dominant contact, the peptide established a cooperative network in which hydrogen bonds likely provide positional specificity and initial anchoring, whereas hydrophobic contacts contribute to interfacial packing and energetic consolidation (Patil et al. 2010). This balance is particularly relevant for peptides derived from PPIs, because interfacial recognition generally requires both geometric complementarity and sufficient solvent shielding to stabilize favorable contacts at the binding surface (Chen and Zacharias 2023). Therefore, the mixed polar/hydrophobic binding mode observed here is consistent with a plausible recognition pattern for an interface‐derived peptide, rather than with a binding event driven exclusively by a catalytic motif. Importantly, this interpretation also agrees with previous studies showing that interface‐derived or docking‐guided peptides targeting lepidopteran trypsins can preserve key recognition determinants associated with inhibitory binding when they preserve the main energetic determinants of the parent protein interaction (Paulo et al. 2026; De Almeida Barros et al. 2021).

The residues identified on the trypsin surface further suggest that PEP‐11 is recognized through a localized binding environment in which both polar and hydrophobic microdomains contribute to peptide accommodation. From a discussion standpoint, the important point is not simply which residues interact, but that the receptor appears to provide a chemically complementary surface able to support both orientation and retention of the peptide. This is relevant because insect digestive trypsins are established targets for pest control, and peptides that engage complementary surface features may provide a rational basis for developing candidates suitable for subsequent selectivity optimization, while retaining the practical advantages of smaller and more tractable molecules than the parent inhibitor protein (Paulo et al. 2026; Meriño‐Cabrera and de Almeida Oliveira 2022). At the same time, these results should still be interpreted as evidence of favorable recognition rather than definitive proof of inhibitory efficacy, which will ultimately depend on experimental validation of enzyme inhibition and selectivity.

The dynamic behavior of the trypsin–PEP‐11 complex is consistent with a binding process in which the initial docked pose undergoes structural adjustment before reaching a more favorable bound ensemble. In protein–peptide systems, this type of early relaxation is commonly interpreted as a consequence of induced‐fit effects and local conformational accommodation, rather than as evidence of instability (Csermely et al. 2010). From that perspective, the subsequent maintenance of a relatively constrained trajectory suggests that the docked pose was not simply retained by geometric coincidence but was refined into a dynamically compatible binding mode. The lower motional amplitude of PEP‐11 relative to the whole complex is also relevant, because peptides that remain conformationally restricted after accommodation are generally more consistent with stable interfacial recognition than peptides that continue to sample broad, weakly bound states. This interpretation agrees with previous studies showing that electrostatic steering and receptor flexibility are central to peptide recognition and that many peptide–protein complexes display an initial adaptation phase followed by stabilization once favorable contacts are optimized (Falkenstein et al. 2023; Dagliyan et al. 2011)

The RMSF pattern further supports the view that PEP‐11 binding is accommodated through localized plasticity rather than through widespread structural perturbation of the enzyme. For a digestive serine protease, this is an important distinction: a candidate peptide does not need to rigidify the entire receptor to be meaningful, but it should be able to engage the binding surface without disrupting the integrity of the overall fold (Chan et al. 2024; Vogt et al. 2014). In this case, the concentration of mobility in terminal and exposed loop regions is compatible with the idea that the receptor preserves a stable structural core while allowing limited surface rearrangements that facilitate peptide accommodation. Such behavior is in line with broader models of protein–peptide recognition, in which side‐chain adjustments and flexible peripheral segments absorb much of the conformational adaptation required for binding, whereas the central scaffold remains comparatively conserved (Linker et al. 2023; Sheehan et al. 2021).

This interpretation is also consistent with previous work on Lepidopteran digestive proteases, where small designed peptides have shown favorable interaction profiles when they preserve the main recognition determinants needed for trypsin‐like enzyme binding. In a pest *Anticarsia gemmatalis*, for example, rationally designed peptides established critical hydrogen‐bonding patterns with trypsin‐like proteases and were subsequently confirmed as competitive inhibitors in vitro, while more recent work has likewise shown that the affinity of short peptides can depend on the balance among hydrogen‐bonding, alkyl/π‐alkyl, and other weak interactions at the binding interface. Within that context, the present dynamic profile does not by itself prove inhibition, but it does strengthen the mechanistic plausibility of PEP‐11 as a trypsin‐targeting candidate: the complex appears to reach a stable bound state without requiring major deformation of the receptor, which is precisely the kind of behavior expected for an interface‐derived peptide intended to preserve recognition while minimizing structural complexity (Paulo et al. 2026; De Almeida Barros et al. 2021).

The combined Rg and SASA behavior suggests that peptide binding is accommodated without large‐scale restructuring of the receptor, which is an important point for interpreting the stability of the trypsin–PEP‐11 complex. In protein–peptide systems, relatively stable Rg values are generally associated with preservation of global compactness, whereas limited SASA variation is more consistent with local surface rearrangements than with extensive unfolding or collapse (Nezhad et al. 2026; Bagewadi et al. 2023). In other words, the complex appears to undergo conformational adjustment at the interface while maintaining the overall architecture of the enzyme, a behavior that is typically expected when binding is driven by surface recognition rather than by deep structural reorganization (Kuzu et al. 2025; Qing et al. 2022). This interpretation is in line with broader molecular dynamics analyses of biomolecular complexes, where stable Rg and SASA profiles are commonly taken as indicators that the bound state remains structurally coherent throughout the simulation window.

The progressive increase in intermolecular hydrogen bonding is also mechanistically informative. Rather than indicating a static lock‐and‐key interaction from the outset, this pattern is more consistent with gradual optimization of the bound state, in which the peptide refines its position within the receptor surface and consolidates more persistent contacts over time (Nagornova et al. 2012). For interface‐derived peptides, such behavior is particularly relevant because binding often depends on a balance between early electrostatic recognition and later stabilization through a more optimized contact network. Similar interpretations have been reported in other protein–peptide systems, where increasing hydrogen‐bond persistence during simulation has been associated with improved accommodation of the ligand and greater stabilization of the complexed state (Paulo et al. 2026 Saleem et al. 2026; Brylinski 2018). In this sense, the hydrogen‐bond profile of trypsin–PEP‐11 supports the idea that the peptide does not merely remain transiently associated with the enzyme surface but evolves toward a more favorable interfacial arrangement.

The MM/GBSA results reinforce this interpretation by showing that the bound state is energetically favorable across the three independent simulations. From a discussion standpoint, the key point is not simply that the calculated ΔGvalues are negative, but that the energetic pattern is reproducible across replicas and remains compatible with the interaction network inferred from docking and trajectory analyses. The limited variation in the electrostatic and van der Waals components suggests that PEP‐11 does not depend on a highly fragile or replica‐specific contact arrangement but instead samples a relatively consistent energetic solution. At the same time, MM/GBSA should be interpreted cautiously: it is most useful here as a comparative and rescoring framework that supports the plausibility of the bound pose, rather than as a definitive predictor of experimental affinity. Even so, this level of energetic consistency is encouraging and compares favorably with the logic used in other peptide‐design studies, including work on lepidopteran gut trypsin‐like enzymes, where small peptides with stable binding profiles have been proposed as promising leads for further biological evaluation (Wang et al. 2020).

Taken together, the docking, molecular dynamics, and MM/GBSA results provide computational evidence of a plausible and energetically favorable binding mode for PEP‐11 on the *S. frugiperda* trypsin model. These findings support the prioritization of PEP‐11 as an interface‐derived peptide candidate for further study. However, they should not be interpreted as direct evidence of functional inhibition or biological selectivity. These properties remain to be established through enzymatic inhibition assays, selectivity studies against related digestive and non‐target proteases, proteolytic stability evaluation, and bioactivity assays in insect models.

An important limitation of the present study is that selectivity was not directly assessed against non‐target proteases or alternative digestive serine proteases. Therefore, although the interaction profile of PEP‐11 is consistent with favorable recognition of the S. frugiperda trypsin model, the current data do not yet establish whether this peptide preferentially recognizes the target enzyme over structurally related off‐target proteases. This point is particularly relevant for the future development of peptide‐based bioinsecticides, since practical applicability will depend not only on binding favorability, but also on the extent to which target recognition can be differentiated from non‐target protease interactions.

## 5 Conclusion

This study demonstrates that the Dioscorin–trypsin protein–protein interface can serve as a rational structural template for the in silico discovery of bioinsecticidal peptides targeting digestive trypsins from *S. frugiperda*. Within this framework, PEP‐11 was prioritized as an interface‐derived peptide scaffold, supported by coherent docking interactions, sustained dynamic stability, and favorable binding energetics across independent simulations. Beyond identifying a single peptide candidate, the principal contribution of this work is the establishment of a structure‐guided pipeline for extracting and computationally prioritizing short inhibitory sequences from biologically relevant protein–protein interfaces, thus providing a rational basis for peptide‐based bioinsecticide discovery. At the same time, these findings remain within the interpretive limits of an in silico study, since inhibitory potency, future selectivity assessment, proteolytic stability, and performance under physiologically and agriculturally relevant conditions have yet to be demonstrated experimentally. Accordingly, future work should integrate in vitro enzymatic assays, selectivity analysis, and peptide optimization aimed at improving stability and bioavailability, followed by in vivo assessment in *S. frugiperda* to determine the translational potential of the computationally prioritized scaffold identified here.

## Author Contributions

**José Severiche‐Castro:** conceptualization, investigation, funding acquisition, writing – original draft, methodology, supervision, writing – review and editing. **Maria Fernanda Valerio:** methodology, validation, writing – original draft, investigation. **Maria Goreti de Almeida Oliveira:** investigation, conceptualization, writing – review and editing, methodology.

## Conflicts of Interest

The authors declare no conflicts of interest.

## Data Availability

The data that support the findings of this study are available from the corresponding author upon reasonable request.
